# Organic metal chalcogenide-assisted metabolic molecular diagnosis of central precocious puberty†

**DOI:** 10.1039/d3sc05633c

**Published:** 2023-11-27

**Authors:** Dan Ouyang, Chuanzhe Wang, Chao Zhong, Juan Lin, Gang Xu, Guane Wang, Zian Lin

**Affiliations:** a Ministry of Education Key Laboratory of Analytical Science for Food Safety and Biology, Fujian Provincial Key Laboratory of Analysis and Detection Technology for Food Safety, College of Chemistry, Fuzhou University Fuzhou Fujian 350108 China zianlin@fzu.edu.cn; b State Key Laboratory of Structural Chemistry, Fujian Institute of Research on the Structure of Matter, Chinese Academy of Sciences (CAS) Fuzhou Fujian 350002 China gewang@fjirsm.ac.cn; c Department of Cardiology, Fujian Provincial Governmental Hospital Fuzhou 350003 China

## Abstract

Metabolic analysis in biofluids based on laser desorption/ionization mass spectrometry (LDI-MS), featuring rapidity, simplicity, small sample volume and high throughput, is expected to be a powerful diagnostic tool. Nevertheless, the signals of most metabolic biomarkers obtained by matrix-assisted LDI-MS are too limited to achieve a highly accurate diagnosis due to serious background interference. To address this issue, nanomaterials have been frequently adopted in LDI-MS as substrates. However, the “trial and error” approach still dominates the development of new substrates. Therefore, rational design of novel LDI-MS substrates showing high desorption/ionization efficiency and no background interference is extremely desired. Herein, four few-layered organic metal chalcogenides (OMCs) were precisely designed and for the first time investigated as substrates in LDI-MS, which allowed a favorable internal energy and charge transfer by changing the functional groups of organic ligands and metal nodes. As a result, the optimized OMC-assisted platform satisfyingly enhanced the mass signal by ≈10 000 fold in detecting typical metabolites and successfully detected different saccharides. In addition, a high accuracy diagnosis of central precocious puberty (CPP) with potential biomarkers of 12 metabolites was realized. This work is not only expected to provide a universal detection tool for large-scale clinical diagnosis, but also provides an idea for the design and selection of LDI-MS substrates.

## Abstract 2

Cu(SPh–COOH) was precisely designed and for the first time investigated as a LDI-MS substrate with enhanced energy transfer and ionization efficiency. A high-accuracy metabolic molecular diagnosis of CPP was achieved using the Cu(SPh–COOH)-assisted LDI-MS platform.

## Graphical abstract

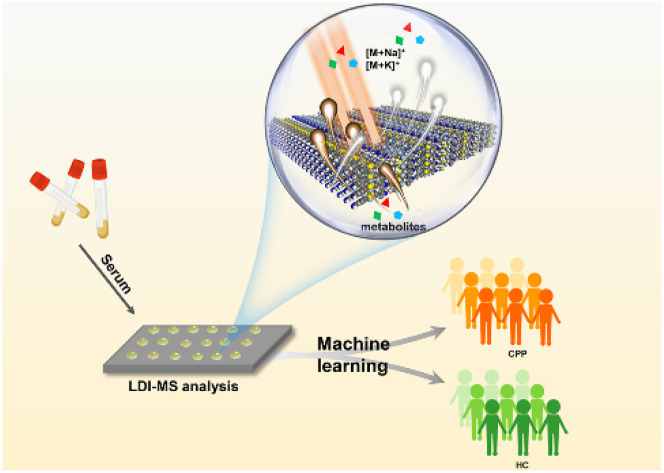

## Introduction

Metabolomics based on laser desorption/ionization mass spectrometry (LDI-MS) plays an increasingly important role in disease diagnosis and discovery of potential biomarkers.^1–6^ Its superiority is mainly reflected in the following aspects: (1) compared to genomics and proteomics, metabolomics reflects the real-time dynamics of disease and is closer to the disease phenotype; (2) LDI-MS is a soft ionization technique with high throughput and salt tolerance, so that only simple dilution or no tedious pretreatment is usually required in the detection of biofluids, which is attractive in large-scale clinical screening; (3) the use of suitable nanomaterials as substrates can eliminate the background interference and ion suppression of traditional matrices in the small molecular region of *m*/*z* < 600, which is a typical molecular weight region for many typical metabolic biomarkers, thus facilitating the analysis of metabolites. However, direct metabolic analysis of biofluids with the low abundance of most metabolites and great sample complexity has proven to be extremely challenging.^7^

Notably, thermal desorption and ion transfer are crucial for the desorption/ionization of analytes in LDI-MS, which depends on the materials used as substrates.^8–10^ Till now, various nanomaterials have been used as LDI-MS substrates for metabolite analysis, such as metals, metal oxide materials, carbon nanotubes, *etc.*^11–16^ Recently, materials of conjugated polymers have attracted increasing attention as substrates for LDI-MS. On the one hand, the large π-system enables them to absorb UV well and therefore facilitates photothermal conversion and energy transfer. On the other hand, the high molecular weight and chemical stability effectively reduce the substrate-related interference peaks.^17–19^ However, the current development of substrates is regularly done by the “trial-and-error” approach, which is often time-consuming, laborious and inefficient.^20,21^ In general, an ideal substrate is expected to exhibit favorable UV absorption and high photothermal conversion efficiency with no background interference.^22,23^ And these are greatly affected by the morphological and surface functionalization of the material.^19,21,24^ Consequently, the precise design of promising materials that can enhance the sensitivity and serve as ideal novel substrates in the low mass range is crucial for metabolic analysis of biofluids in LDI-MS.

Organic metal chalcogenides (OMCs), a new class of two-dimensional (2D) organic-inorganic hybrid materials in which the metal chalcogenide layers are covered by organic functional groups through covalent bonding, have been adopted in photoelectricity, sensors, photothermal therapy and energy transfer due to their unique electronic properties, high UV absorption, and appreciable energy transfer capability.^25,26^ The high designability of both the inorganic layers and the organic functional elements endows OMCs with tunable optical absorption ability, carrier type, carrier mobility, *etc.*, which contributes to high photothermal conversion and appreciable efficient ion transfer.^27^ In addition, OMCs can be better dispersed in various solvents with the inorganic layer completely covered by organic functional groups, which is beneficial to obtain a uniformly dispersed substrate solution and therefore improves the sensitivity and reproducibility of LDI-MS.^28,29^ Therefore, OMCs show great prospects as a new category of functional high performance substrates for LDI-MS. Interestingly, to our knowledge, the potential of OMCs as substrates for LDI-MS has never been explored before.

Herein, four few-layered OMCs with high designability were synthesized *via* a strategy of organic modification followed by exfoliation and investigated as novel substrates for promising candidates of LDI-MS for the first time. The properties of the four few-layered OMCs were studied from the perspectives of UV absorption, energy transfer and charge transport to further explore the desorption/ionization mechanisms. The introduction of a large number of benzene ring structures endowed the four OMCs with high UV absorption. In addition, by adjusting functional groups and metal nodes, the internal energy transfer and ionization efficiency within the system were greatly enhanced. Finally, Cu(SPh–COOH), the OMCs with Cu^+^ as the metal node and –COOH as the functional group was optimized to be the LDI-MS substrate with greatly enhanced signal response, clean background and high salt and protein tolerance. As a result, several typical metabolites and different saccharides with the molecular weight range from 180 to 828 Da were successfully detected. Moreover, due to the excellent desorption/ionization ability, Cu(SPh–COOH) assisted the highly accurate diagnosis of central precocious puberty (CPP) based on the serum metabolic fingerprint (SMF).

## Results and discussion

### Synthesis and characterization of few-layered OMCs

**Fig. 1:**
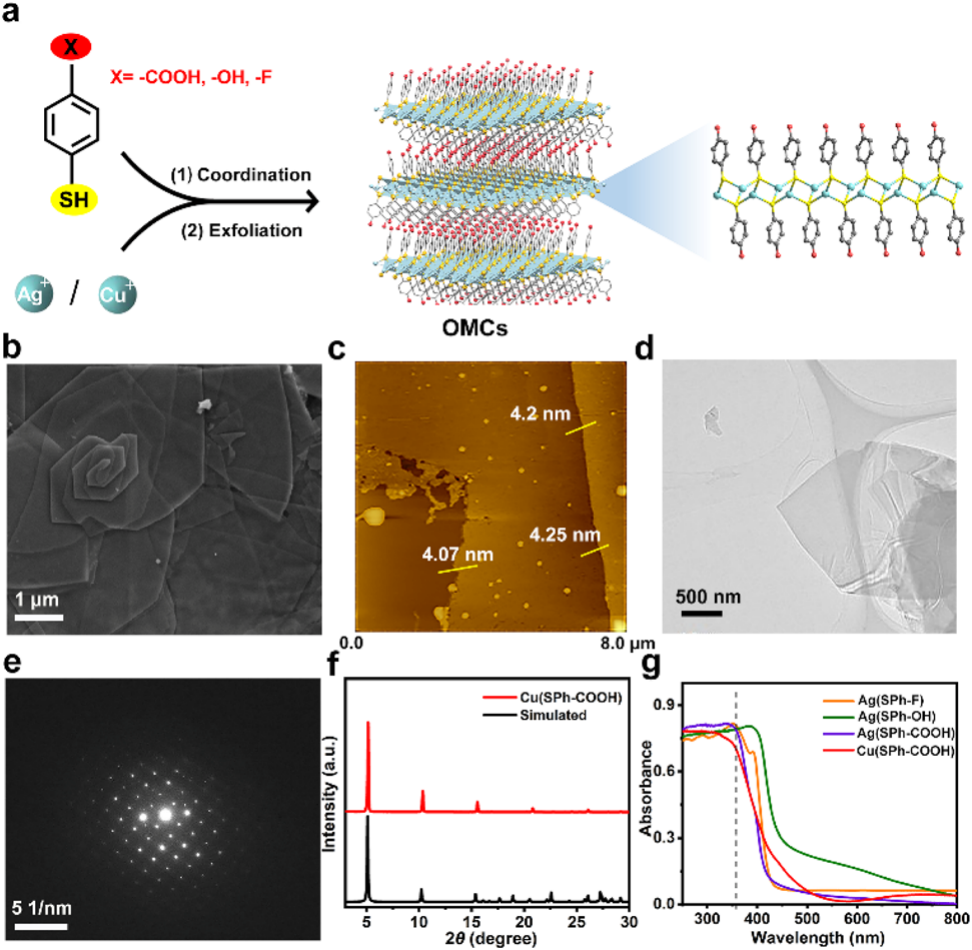
(a) Scheme of preparation for few-layered OMCs; (b) SEM image, (c) AFM image, (d) TEM image, (e) corresponding SAED pattern, and (f) PXRD pattern of Cu(SPh–COOH) nanosheets; (g) UV-vis spectra of the four few-layered OMCs (the dotted line represented the laser wavelength (355 nm) used for LDI-MS).

The few-layered OMCs were synthesized by an organic modification and then exfoliation strategy and named M(SPh–X) (M represents Cu^+^ or Ag^+^, HSPh represents benzenethiol, and X represents –COOH, –OH, or –F in organic ligands), bearing not only ordered and abundant functional groups in both surfaces, but also tunable functional groups and metal ions (Fig. 1a).^25^ The scanning electron microscopy (SEM) images in Fig. 1b and S1† illustrated M(SPh–X) with laminar architectures. The atomic force microscopy (AFM) images and transmission electron microscopy (TEM) images showed the nature of M(SPh–X) as 2D nanosheets (Fig. 1c and d, S2, and S3†) with the thicknesses of approximately 4–6 nm. The selected area electron diffraction (SAED) and powder X-ray diffraction (PXRD) patterns in Fig. 1e and f and S4† confirmed the crystal structures of OMCs. The PXRD patterns were in agreement with the simulated patterns, suggesting the successful synthesis of these few-layered OMCs. It is well known that promising optical properties are one of the prerequisites for being the substrate of LDI-MS. As displayed in Fig. 1g, these nanosheets all exhibited strong UV absorption in 355 nm (the emission wavelength of the laser source), showing great potential as substrates for LDI-MS. In addition, the four nanosheets were found to have good solvent dispersibility (Fig. S5†), which was favorable to improve the sensitivity in LDI-MS.

### Performance of few-layered OMCs for LDI-MS

**Fig. 2:**
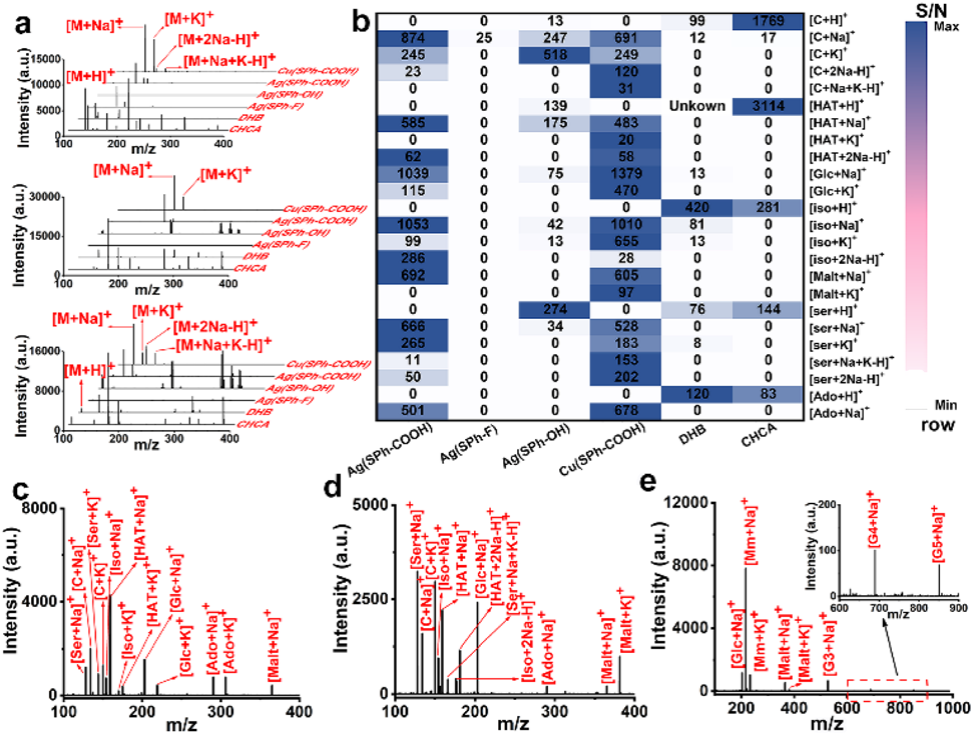
(a) Mass spectra of Iso, Glc and Ser (from top to bottom) and (b) the heatmap showing the S/N of seven metabolites with different matrices consisting of the four few-layered OMCs, CHCA, and DHB; mass spectra of the mixture of (c) seven metabolites, (d) seven metabolites in the presence of 0.5 M NaCl and 5 mg per mL BSA, and (e) six saccharides with Cu(SPh–COOH) as the substrate.

Seven small metabolites including serine (Ser), cytosine (C), isoleucine (Iso), hypoxanthine (HAT), glucose (Glc), adenosine (Ado), and maltose (Malt) were selected to assess the performance of the four few-layered OMCs as substrates (Fig. 2a and S6†). For functional group selection, when Ag^+^ was used as the metal node, Ag(SPh–COOH) afforded the best performance as a substrate with a cleaner background and higher MS signal at a laser intensity of ∼388 μJ mm^−2^ and also performed better than organic matrices including α-cyano-4-hydroxycinnamic acid (CHCA) and 2,5-dihydroxybenzoic acid (DHB). Ag(SPh–OH) could detect some of these metabolites with serious background interference, while Ag(SPh–F) showed almost no response. On the other hand, the metal node was further changed to Cu^+^ and the result manifested that Cu(SPh–COOH) performed as well as Ag(SPh–COOH), with about 10 000 times higher signal intensity than the other two OMCs. Notably, the laser intensity of Cu(SPh–COOH) was ∼414 μJ mm^−2^. In addition, the signal-to-noise ratio (S/N) of the seven metabolites with the above matrices was integrated to make a better comparison, as shown in Fig. 2b, which indicated that the changes of functional groups and metal nodes had a great influence on the performance of the desorption ionization process. It should be emphasized that when Ag(SPh–COOH) was used as the substrate, serious background interference was produced as the laser intensity exceeded 414 μJ mm^−2^ (Fig. S7†). Meanwhile with Cu(SPh–COOH) as the substrate, the background remained clean even when the laser intensity was increased to 465 μJ mm^−2^. This might be due to the fact that the coordination of Cu^+^ and –SH was stronger than that of Ag^+^ and –SH according to soft and hard acid–base theory.^30^ Thus, Cu(SPh–COOH) was finally selected as the substrate for the subsequent detection.

To further verify the analytical ability in complex samples, the abovementioned seven metabolites were mixed for LDI-MS analysis. As seen from Fig. 2c, all the metabolites were observed in the form of Na^+^ or/and K^+^ adduct peaks. Also, the mixture was successfully detected in the presence of high salt and protein concentrations (Fig. 2d), demonstrating the great potential of Cu(SPh–COOH) as a LDI-MS substrate for complex real samples. Furthermore, the limit of detection (LOD) of Ser, C, and Glc was found as low as levels of pg mL^−1^ (Fig. S8†).

As known to all, saccharides are essential metabolites in life activities. However, direct analysis of saccharides confronts significant challenges due to their typically low abundance in complex samples and the lack of UV-absorbing moieties.^31^ Therefore, a mixture of six saccharides including Glc, methyl mannoside, Malt, maltotriose (G3), maltotetraose (G4) and maltopentaose (G5) with the molecular weight range from 180 to 828 Da was analyzed. As presented in Fig. 2e, all five saccharides were successfully detected, indicating that the substrate had a positive ability to promote desorption/ionization of analytes. In order to further verify the ability of Cu(SPh–COOH)-assisted desorption ionization, a comprehensive comparison was made with several reported nano-substrates (including ZnFe_2_O_4_ nanocrystal clusters, gold nanoparticles (Au), multiwalled carbon nanotubes (MWCNTs) and hydroxylated multiwalled carbon nanotubes (OH-MWCNTs)) in detecting saccharides. By taking malt as the model compound, it was seen from Fig. S9a–e† that Au, MWCNTs and OH-MWCNTs had strong background interference, while Cu(SPh–COOH) presented a clean background and higher mass spectral signal. It is worth noting that Glc was observed in the five substrates due to the small amount of Glc contained in the Malt standard. In addition, G3, G4, and G5 were further evaluated with the five nano-substrates, respectively. The results in Fig. S9f† showed that a higher S/N was obtained with the Cu(SPh–COOH) substrate. In contrast, G4 and G5 were not detected with some of the other four nano-substrates, suggesting that Cu(SPh–COOH) may be able to provide richer information in analyzing complex biological samples.

### Mechanism of Cu(SPh–COOH) in the enhanced desorption/ionization process

**Fig. 3:**
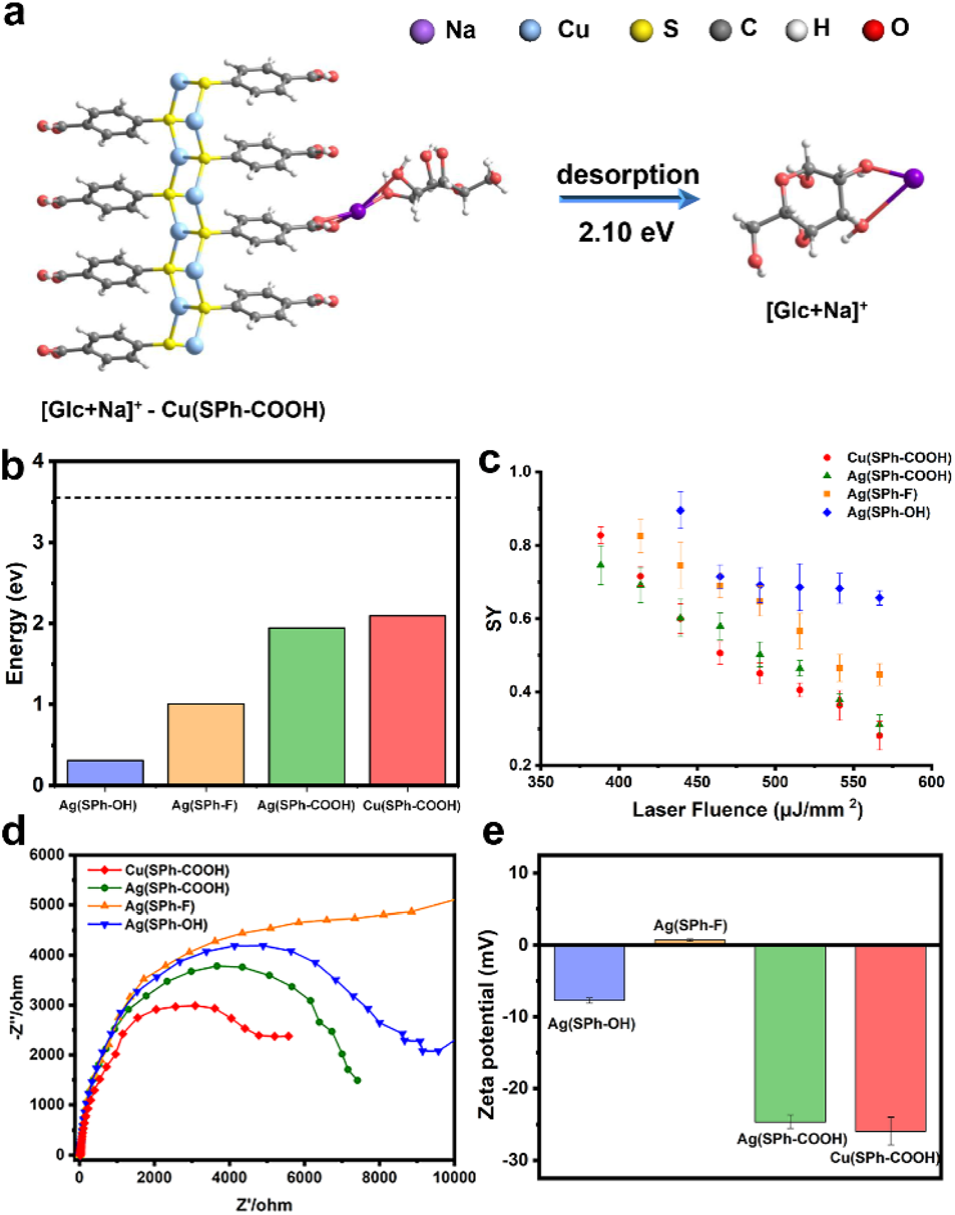
(a) Desorption of [Glc + Na]^+^ from Cu(SPh–COOH) surface simulated by DFT, (b) the desorption energies of [Glc + Na]^+^ from the four OMCs (the dotted line represents the laser energy (3.55 eV) used for LDI-MS), (c) effect of laser fluence on the SY value of 4-MeBP^+^ with the four few-layered OMCs as substrates, and (d) EIS and (e) zeta potential of the four few-layered OMCs.

In order to explore the mechanism of Cu(SPh–COOH) in the enhanced desorption ionization process, we first calculated the energy required for desorption from the surface of the four OMCs with [Glc + Na]^+^ as a model compound. The desorption energies were obtained by simulating the corresponding path based on density functional theory (DFT) in Fig. 3a and S10–S12.† As presented in Fig. 3b, the desorption energies of all four OMCs were lower than that of the laser energy (3.55 eV) for LDI-MS. Therefore, the analyte had the possibility of desorption from the material during laser irradiation. However, previous experimental results indicated that the desorption ionization of Ag(SPh–OH) and Ag(SPh–F) as substrates of LDI-MS was not satisfactory. This is probably due to the interaction between substrates and analytes, where the adsorption energy of Ag(SPh–OH) and Glc was only ∼0.3 eV, making it difficult to form a relatively stable analyte–substrate complex and thus preventing effective heating and desorption.^24,32^ According to the thermally driven desorption, effective thermal energy transfer from the substrate to analytes is of great importance to the desorption ionization process.^33^ Consequently, the 4-methylbenzylpyridinium ion (4-MeBP^+^), a chemical thermometer to indirectly measure the photothermal conversion efficiency of the substrate system, was employed to get a deep insight into the correlation between the internal energy content of analyte ions and desorption/ionization efficiency.^34–36^ 4-MeBP^+^ was subject to simple fragmentation in LDI-MS with the generation of neutral pyridine (Py) and the 4-methylbenzyl cation (4-MeB^+^) (Fig. S13†).4-MeBP^+^ → 4-MeB^+^ + Py

Then the survival yield (SY) method helped to understand the extent of internal energy transfer in LDI-MS. The SY value was calculated by using the formula below:1
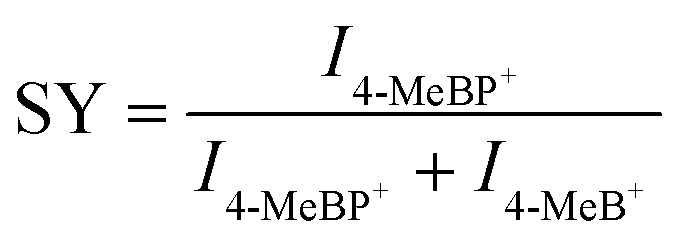
where *I* represents the MS signal intensity. The SY value was in inverse proportion to the internal energy.^37^ As observed in Fig. 3c, the survival of 4-MeBP^+^ decreased with the laser pulse energy increased due to an increase in the energy absorbed by the substrate and an increase in the energy transferred to the analyte. The lowest SY value was obtained with M(SPh–COOH) as the substrate, implying that M(SPh–COOH) might be able to perform better photothermal conversion and internal energy transfer. Meanwhile Ag(SPh–OH) required a higher laser fluence threshold and exhibited a relatively higher SY value, suggesting that this material was not effective in transferring energy to the analyte, which might be the reason for its poor desorption ionization. Notably, Ag(SPh–F) failed to serve as a satisfactory substrate although it exhibited certain energy transfer ability. This might be related to its charge separation and transport capability. As shown in Fig. S14† and 3d, photoelectrochemical analysis and electrochemical impedance spectroscopy (EIS) of the four OMCs were performed, where Ag(SPh–F) exhibited almost no photocurrent response and the largest Nyquist semicircle radius, which confirmed our hypothesis. In contrast, Cu(SPh–COOH) had a significantly enhanced photocurrent response and the smallest Nyquist semicircle radius, indicating that it had a reduced electron–hole complexation rate and excellent charge transfer capability, which is conducive to the charge transfer during LDI-MS detection and improves the detection sensitivity.^38^

Interestingly, LDI-MS measurements were not allowed in negative ion mode with all four OMCs. In general, –COOH is more likely to protonate the analyte to be detected in positive ion mode. For –OH, it was shown that small molecule analytes tended to be adsorbed to hydroxyl-rich surfaces through hydrogen bonding and van der Waals forces, followed by laser energy transfer to initiate the desorption ionization process.^34,39,40^ Besides, the zeta potential measurements were performed as shown in Fig. 3e, and the zeta potentials of M(SPh–COOH) were between −22 and −18 mV, which contributed to the ionization efficiency of small molecules in positive ion mode by binding to Na^+^/K^+^ through electrostatic interaction during ionization.^1,6^

### Diagnosis of CPP by Cu(SPh–COOH)

Precocious puberty (PP), one of the most common endocrine disorders in children, refers to the abnormal early appearance of signs of pubertal development before the age of 8 years in girls and 9 years in boys, which is correlated with the negative health consequences in adulthood, including obesity, diabetes mellitus type 2, cardiovascular disease and breast cancer.^41^ However, with improvements in health, nutrition and hygiene, the incidence of PP increases and the age of onset is earlier.^42^ In addition, central precocious puberty (CPP), the most common phenotype (accounting for 80% of PP), has rarely been studied separately.^43^ Currently, the gonadotropin releasing hormone (GnRH) stimulation test is still the gold standard in the diagnosis of CPP, which is time-consuming and distressing.^44^ Therefore, there is an urgent need to find a simpler diagnostic modality for CPP.

**Fig. 4:**
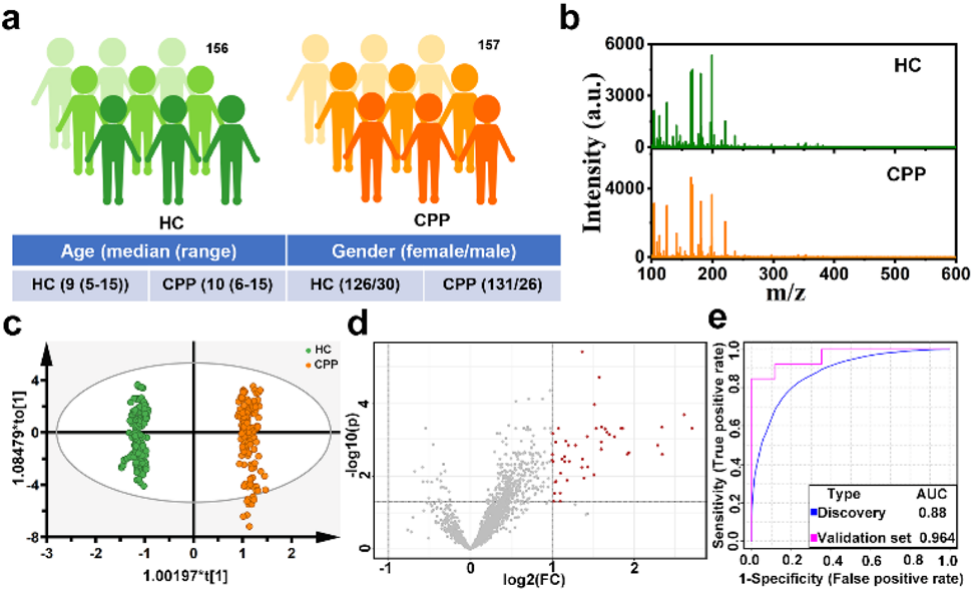
(a) Demographics of patients and healthy controls. (b) Typical mass spectra of serum from CPP and HC, (c) OPLS-DA score plots, (d) volcano plots of metabolites and (e) ROC curves based on the key *m*/*z* for distinguishing CPP and HC.

Encouraged by the excellent performance of Cu(SPh–COOH) as a substrate, we attempted to extract serum metabolic fingerprinting (SMF) from CPP with the Cu(SPh–COOH)-based LDI-MS platform. Primarily, a power analysis was carried out to calculate the sample size required for enabling meaningful machine learning. As illustrated in Fig. S15,† with a predicted power of 0.8 and a false discovery rate (FDR) of 0.1, the resulting minimum sample size was ∼260 (130/130, CPP/HC). Subsequently, a total of 313 serum samples were collected including 156 healthy controls (HC) and 157 CPP (Table S1†). In addition, the one-way analysis of variance (ANOVA) and Chi-square test revealed no significant difference in the age and gender between the two groups (*P* > 0.05). In terms of gender, the prevalence of girls is much higher than that of boys (Fig. 4a and Table S1†).^43^ Without tedious pretreatment, serum samples of HC and CPP were analyzed by Cu(SPh–COOH)-assisted LDI-MS and the SMF is represented in Fig. 4b with rich peaks. Firstly, orthogonal partial least squares discriminant analysis (OPLS-DA) was adopted to classify CPP and HC, in which the two groups were clearly isolated (*R*^2^*Y*(cum) = 0.991 and *Q*^2^(cum) = 0.99) in Fig. 4c, indicating that the SMF of CPP differed significantly from those of HC. Moreover, the results of 200 permutations in Fig. S16† reflected that the model was not over fitted. Furthermore, the volcano plots were utilized to study the differential expression of SMF between CPP and HC. As revealed in Fig. 4d, the red dots represented the up-regulation signals in CPP patients, obtained under the conditions of fold change > 2/fold change < 0.5 and *p*-value (*t*-test) < 0.05. After that, 43 *m*/*z* features were screened as potential metabolic biomarkers in combination with the variable importance in the projection (VIP) value > 1.5 obtained from the OPLS-DA model. Then, 20% samples of the two groups were randomly categorized as the validation cohort and 80% as the discovery cohort to verify the classification performance of the model. As a result, a receiver operating characteristic (ROC) curve based on the above 43 *m*/*z* features is shown in Fig. 4e, with an area under the curve (AUC) of 0.88 from the discovery cohort and 0.964 from the validation cohort, further revealing that the screened key *m*/*z* had potential as a biomarker.

**Fig. 5:**
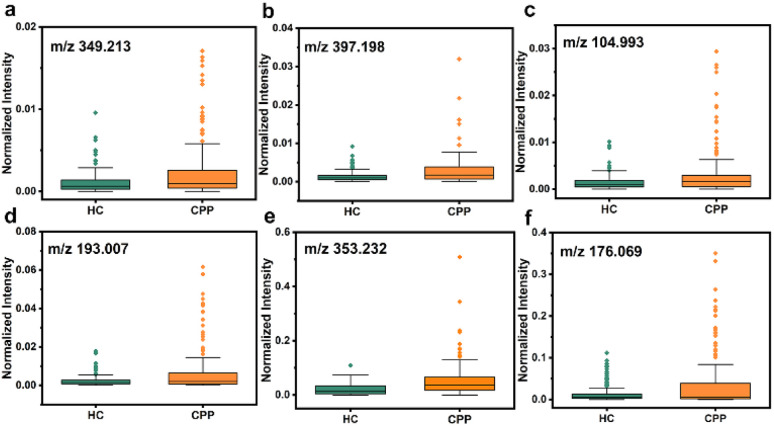
Dysregulation of six potential biomarkers in serum samples between HC and CPP. (a) Arachidonic acid, (b) PGE2, (c) acetic acid, (d) citramalic acid, (e) 9,10,13-TriHOME, and (f) dopamine.

Therefore, metabolites were further searched manually in the Human Metabolome Database with a molecular weight tolerance of ±10 ppm in an attempt to identify certain molecules associated with disease. Eventually, 12 well-matched *m*/*z* were defined as potential biomarkers of CPP and their detailed information is listed in Table S2.† As seen in Fig. 5 and S17,† all 12 metabolites were significantly up-regulated in the disease group compared to HC. Then, two *m*/*z* with high signal intensity were selected for MS/MS analysis as the method verification (Fig. S18†). The results were consistent with the substances identified by the accurate mass measurement, indicating the feasibility of the method. Docosapentaenoic acid (22n-3) is an essential omega-3 fatty acid that is involved in the metabolism of alpha-linolenic acid. Besides, citramalic acid, 9,10,13-trihydroxy-11-octadecenoic acid (9,10,13-TriHOME), pregnanetriol, and (3beta,22*E*,24*R*)-3-hydroxyergosta-5,8,22-trien-7-one are related to the chemical reaction of lipid peroxidation. These metabolites may participate in the formation of CPP by being involved in the regulation of lipid metabolism, suggesting that the CPP required additional energy for enhanced energy metabolism.^45^ In addition, prostaglandin E2 (PGE2) together with glutathione, arachidonic acid, and 15-hydroperoxy-(5*Z*,8*Z*,11*Z*,13*E*)-eicosatetraenoic acid (15-HPETE) is involved in arachidonic metabolism, as shown by metabolic pathway enrichment analysis in Fig. S19,† which can in turn promote the production of prostaglandins.^46^ PGE2, as the most biologically active and most common prostaglandin, can facilitate luteinizing hormone-releasing hormone (LHRH) secretion, which stimulates the production of gonadotropin, and impacts on sexual development by interacting with sex hormones.^47^ Therefore, based on the above analysis, PGE2 can be one of the significant factors to distinguish between HC and CPP patients. On the other hand, upregulation of acetic acid, dopamine and serotonin indicated the activation of the hypothalamic–pituitary gonadal axis (HPGA), which triggers early puberty.^48^

## Conclusions

In summary, several high-performance OMCs were precisely designed and introduced for the first time as substrates for LDI-MS. In this way, a high-accuracy metabolic molecular diagnosis platform based on OMC-assisted LDI-MS was developed, which achieved efficient diagnosis of CPP with the advantages of small sample consumption, simple sample processing, excellent salt and protein tolerance and high throughput. Moreover, by changing the metal nodes and organic functional groups of the OMCs, saccharides with the molecular weight range from 180 to 828 Da were successfully detected. The mechanism investigation suggested that this might be mainly ascribed to the following reasons: (1) the alteration of the OMC structure allowed higher energy to be transferred to the analytes for desorption from the substrate; (2) favorable charge separation and transport capability promoted the desorption ionization process; (3) the introduction of acidic organic groups made the surface of OMCs negatively charged, which promoted the ionization process in the positive ion mode. In addition, interactions between substrate and analyte molecules might affect energy transfer. It is to be noted that the change of the metal node from Ag^+^ to Cu^+^ enhanced the stability of OMCs and showed less background interference in LDI-MS. This work is expected to solve the clinical needs of disease diagnosis, and in addition, offers ideas to unravel the mechanisms of the LDI-MS substrate, which is more conducive to systematically and logically designing effective substrates than the traditional continuous trial-and-error approach.

## Data availability

The datasets supporting this article have been uploaded as part of the ESI.†

## Author contributions

Dan Ouyang: investigation, conceptualization, data curation, visualization, and writing – original draft preparation. Chuanzhe Wang: investigation, data curation, and writing – reviewing and editing. Chao Zhong: writing – reviewing and editing. Juan Lin: resources. Gang Xu: conceptualization and writing – reviewing and editing. Zian Lin and Guane Wang: conceptualization, writing – reviewing and editing, and funding acquisition. Dan Ouyang and Chuanzhe Wang contributed equally to this work.

## Conflicts of interest

There are no conflicts to declare.

## Supplementary Material

SC-015-D3SC05633C-s001
