# Supplementary material for: Organic metal chalcogenide-assisted metabolic molecular diagnosis of central precocious puberty
Source: Chem Sci. 2023 Nov 27;15(1):278–84. doi: 10.1039/d3sc05633c (PMC10732007; doi:10.1039/d3sc05633c)
Supplement: SC-015-D3SC05633C-s001 [file SC-015-D3SC05633C-s001.pdf]

Electronic Supplementary Material (ESI) for Chemical Science.
This journal is © The Royal Society of Chemistry 2023

Supporting Information

Organic Metal Chalcogenides-Assisted Metabolic Molecular Diagnosis of Central Precocious Puberty

Dan Ouyang,+[a] Chuanzhe Wang,+[b] Chao Zhong,[a] Juan Lin,[c] Gang Xu,[b] Guane Wang*[b], and Zian Lin*[a]

[a] Ministry of Education Key Laboratory of Analytical Science for Food Safety and Biology, Fujian Provincial Key Laboratory of Analysis and Detection Technology for Food Safety, College of Chemistry, Fuzhou University, Fuzhou, Fujian, 350116, China

[b] State Key Laboratory of Environmental and Biological Analysis, Department of Chemistry, Hong Kong Baptist University, 224 Waterloo Road, Kowloon Tong, Hong Kong, SAR, P. R. China

[c] Department of Cardiology, Fujian Provincial Governmental Hospital, Fuzhou 350003, China

*Corresponding author: Zian Lin; Guane Wang

E-mail: zianlin@fzu.edu.cn (Z. Lin); gewang@fjirsm.ac.cn (G. Wang).

Experimental Procedures

Chemicals and Materials. 4-Mercaptophenol (97%), 4-Fluorothiophenol (98%), maltotetraose (G4, MW=666.58), 4-methylbenzyl chloride, pyridinium, serotonin (MW=176.22), Dopamine (MW=153.18) and 2,5-dihydroxybenzoic acid (DHB) were purchased from Aladdin. Maltotrise (G3, MW=504.44) was purchased from Macklin. 4-Mercaptobenzoic acid (90%) was purchased from Adamas Reagent, Ltd. China. Silver nitrate (AR, ≥99.8%), Copper (II) chloride dehydrate (AR, ≥99.0%) were purchased from Sinopharm Chemical Reagent Co., Ltd, China; Hydrogen chloride (AR, 36.0-38.0%, w%) was purchased from Sinopharm Chemical Reagent Co., Ltd, China. Serine (Ser, MW=105.09), cytosine (C, MW=111.10), isoleucine (Iso, MW=131.17), hypoxanthine (HAT, MW=136.11), glucose (Glc, MW=180.16), methyl mannoside (MW=194.18), adenosine (Ado, MW=267.24), maltose (Malt, MW=360.31), maltopentaose (G5, MW=828.72), sodium chloride, and bovine serum albumin (BSA) were provided by Sigma-Aldrich. α-Cyano-4-hydroxycinnamic acid (CHCA) was obtained from Bruker Doltonics Crop (Germany). Other reagents were of analytical grade or better. Deionized water (18.2 MΩ cm^{-1}) was produced by Milli-Q water purification system (Millipore, USA).

Synthesis of four OMCs. The OMCs nanosheets were prepared in a similar method by varying the organic ligands and metal ions according to a reported literature with some modifications.[1]

Synthesis of Cu(SPh-COOH) nanosheet. $CuCl_2 \cdot 2H_2O$ (12 mg) was dissolved in 200 mL of ethanol and water (1:1, v/v), then 30 mg 4-Mercaptobenzoic acid was added into the solution, stirred at 100 °C for 6 h, and continued to stir for 12 h after cooling down. Finally, the Cu(SPh-COOH) nanosheet was obtained by centrifugation and washed with ethanol and water, respectively.

Synthesis of Ag(SPh-COOH) nanosheet. 4-Mercaptobenzoic acid (45.45 mg) and $AgNO_3$ (25 mg) were dissolved in 250 mL of water and the mixed solution was sonicated at 400 W for 10 min. Then the solution was kept at a constant temperature in an oven at 100 °C for 24 h to obtain a white suspension and a white precipitate. The product was collected by centrifugation of the upper white suspension at 10,000 rpm and washed sequentially with ethanol and water, and finally dried under vacuum at 65 °C.

Synthesis of Ag(SPh-OH) nanosheet. 25 mg $AgNO_3$ was dissolved in 100 mL of water, then 100 μL 4-hydroxythiophenol was added into the solution. Later, 1 mL of triethylamine was added, stirred at 85 °C for 6 h, and continued to stir for 12 h after cooling down. The Ag(SPh-OH) nanosheet was obtained by centrifugation and washed with ethanol and water, respectively.

Synthesis of Ag(SPh-F) nanosheet. 20 mg of $AgNO_3$ was dissolved in 30 mL acetonitrile and 80 μL of 4-Fluorothiophenol was added dropwise to obtain a milky suspension. Next, 0.5 mL of triethylamine was added dropwise and the

milky suspension was instantly clarified. Finally, it was stirred at room temperature until silver-white nanosheet was obtained. The Ag(SPh-F) nanosheet was collected by centrifugation and washed with ethanol.

Synthesis of 1-(4-Methylbenzyl)pyridinium (4-MeBP) Chloride. According to the previously reported literature,[2] 4-MeBP chloride was synthesized by reacting 4-methylbenzyl chloride with 3 mL of pyridine (1:20, molar ratio) under 60 °C for 5 hours.

Characterization. Scanning electron microscopy (SEM) images were collected by a FEI Quanta 250 scanning electron microscope. Transmission electron microscopy (TEM) images were taken with a Hitachi HT7700. The powder X-ray diffraction (PXRD) patterns were acquired on a X'Pert-Pro MPD (Philips, Holland) power diffractometer. Atomic force microscopy (AFM) images were taken with a Bruker Dimension ICON atomic force microscope. UV-visible absorption spectra of the materials were obtained by UV-Vis spectrum measurements (Agilent Cary 7000). The zeta potential was measured from dynamic light scattering (DLS, Zetasizer NanoZS90, Malvern Instruments Ltd, UK). The photocurrent was carried out in a standard three-electrode system with materials as the working electrode, platinum wire as a counter electrode, Ag/AgCl as a reference electrode, and 0.5 M Na_2SO_4 as electrolyte solution with 30 μL Nafion in a CHI 660E electrochemistry workstation (ChenHua Instruments, Shanghai, China).

Density function theory (DFT) calculation. The critical desorption step of $[Glc+Na]^+$ from the OMCs surface was the prerequisite of $[Glc+Na]^+$ formation and determined whether it could be detected. All calculations were carried out with CP2K package (version 7.1) in the framework of the density functional theory,[3] based on the hybrid Gaussian and plan-wave scheme.[4] Molecular orbitals of the valence electrons were expanded into DZVP-MOLOPT-SR-GTH basis sets,[5] while atomic core electrons are described through Goedecker-Teter-Hutter (GTH) pseudopotentials.[6] A plane-wave density cutoff of 500 Ry was adopted. The long range van der Waals interaction is described by the DFT-D3 approach.[7] All the structures fully relaxed by CP2K with BFGS scheme, and the force convergence criterion was set to 4.5 * 10^{-4} hartree/bhor. The multiplicity of the system was tested until converged. The configuration of $[Glc+Na]^+$ and the preferential orientation of glucose molecule on the OMCs surface referred to the previous literature.[8] In addition, the desorption energy (E_d) was calculated by the following formula:

$E_d = E_{substrate} + E_{adsorbate} - E_{substrate-adsorbate}$ where $E_{substrate-adsorbate}$ represented the energy of whole adsorption model, $E_{substrate}$ and $E_{adsorbate}$ were the energies of substrate and adsorbate.

Sample collection. This study obeyed the Declaration of Helsinki and was approved by the Ethics Committee of Fuzhou Children's Hospital. Serum samples were collected from patients who were diagnosed as central precocious puberty (CCP) according to a clinically strict diagnostic protocol and were not treated before in Fuzhou Children's Hospital (Fuzhou, China). In addition, exclusion criteria were other forms of PP, obesity, chronic diseases, developmental anomalies, medication use, and history of radiotherapy or chemotherapy. Healthy control serum samples were collected from individuals for health examination who were not diagnosed with CPP. The serum samples were stored at -80 °C before use.

Sample Preparation. Serum samples, standard metabolites, and prepared mixtures were dissolved in deionized water, then 1 μL solution spotted on the polish plate and dried at room temperature for subsequent LDI MS detection. With no expensive and time-consuming pretreatments, the serum samples were simply diluted 10-fold for analysis to gather m/z values under 600 Da. 4-MeBP chloride was dissolved in ethanol /water (1:1, v/v) with the concentration of 2 μM. For the matrixes preparation, DHB (10 mg/mL) was dissolved in acetonitrile/ water containing 0.1% TFA (3:7, v/v). CHCA (10 mg/mL) was dissolved in acetonitrile/water containing 0.1% TFA (3:7, v/v).

Mass Spectrometry Analysis. All LDI MS detections were performed on Autoflex MALDI-TOF/TOF (Bruker) with the Nd:YAG laser (2 kHz, wavelength of 355 nm) and smart beam system. The acquisitions were carried out in positive ion reflector mode and the MS/MS was in LIFT MS/MS positive mode. MS spectra were generated at 1 kHz and 20 kV with the delay time of 250 ns and the number of laser shots of 300. The program was pre-calibrated by standard molecules. All spectra were used directly without any smoothing procedures.

Statistical analysis. A total of 313 individuals were divided into CPP group (157 serum samples from CPP patients) and control group (156 serum samples from healthy individuals). No significant difference was found in age among controls and CPP patients by one-way ANOVA performed on SPSS (version 19.0, SPSS Inc., Chicago). The raw data of mass spectra was extracted in Flexanalysis 3.4. The model of OPLS-DA based on paretovariance (Par) scaling and the permutation tests with 200 iterations were established by SIMCA (version 14.0, Umetrics, Umeå, Sweden). The ROC curves were established at Metaboanalyst 5.0 (McGill University, Montreal, Canada, https://www.metaboanalyst.ca/). The searching of potential biomarkers was performed with the limitation of 10 ppm in Human Metabolome Database (https://hmdb.ca/).

Supplementary Figures

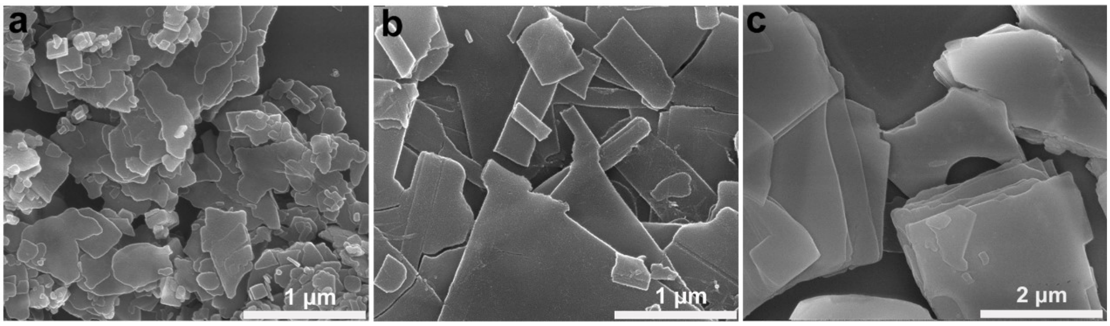

Figure S1. SEM of (a) Ag(SPh-COOH), (b) Ag(SPh-OH), (c) Ag(SPh-F).

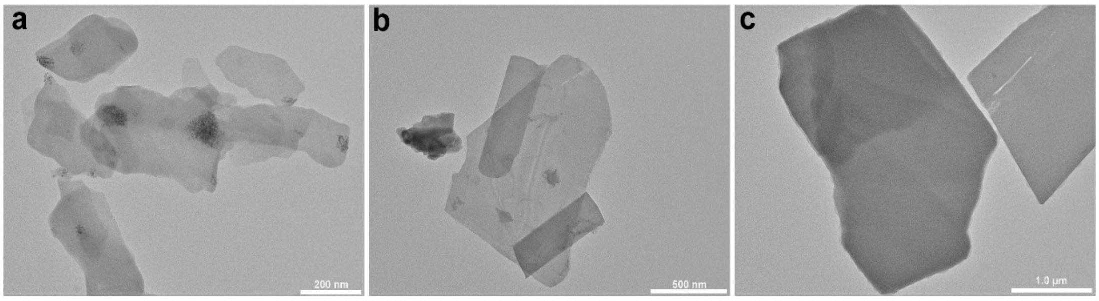

Figure S2. TEM of (a) Ag(SPh-COOH), (b) Ag(SPh-OH), (c) Ag(SPh-F).

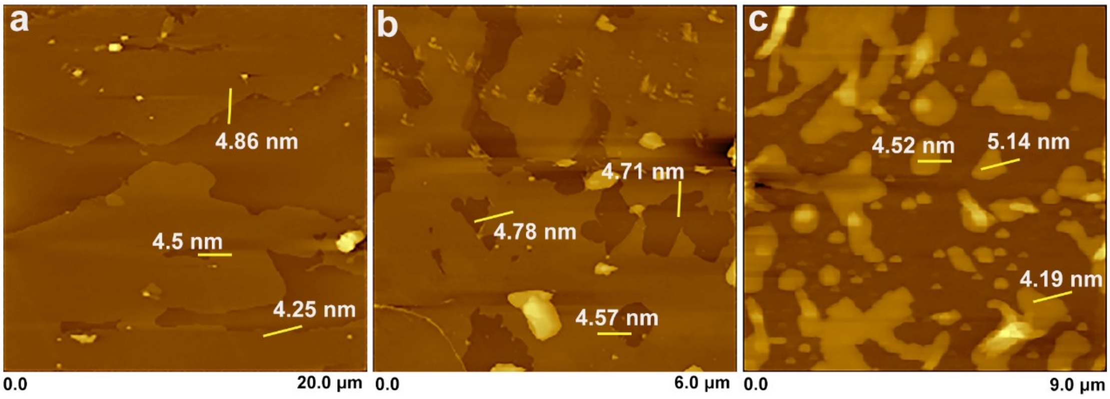

Figure S3. AFM of (a) Ag(SPh-COOH), (b) Ag(SPh-OH), (c) Ag(SPh-F).

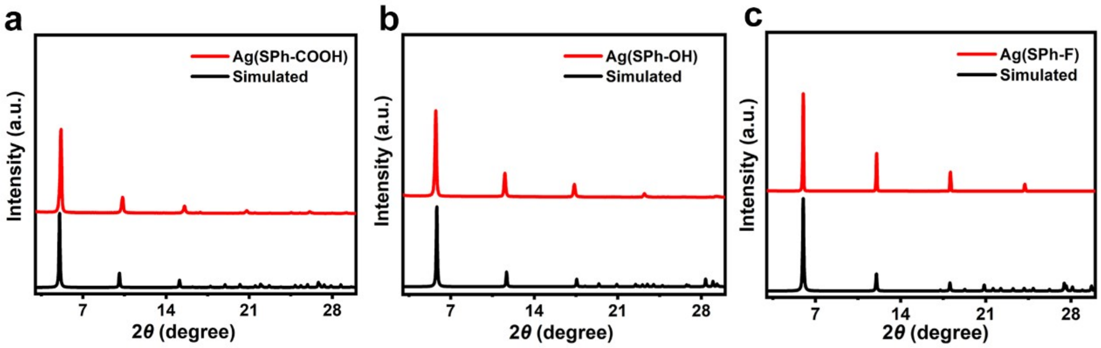

Figure S4. PXRD patterns of (a) Ag(SPh-COOH), (b) Ag(SPh-OH), (c) Ag(SPh-F).

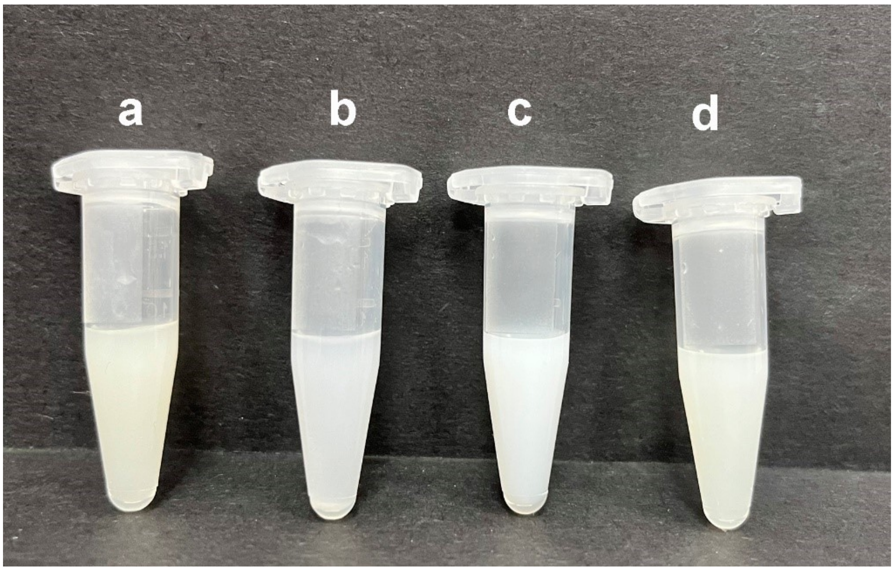

Figure S5. The dispersibility of (a) Cu(SPh-COOH), (b) Ag(SPh-F), (c) Ag(SPh-COOH), and (d) Ag(SPh-OH) in ethanol and water (1:1, v/v).

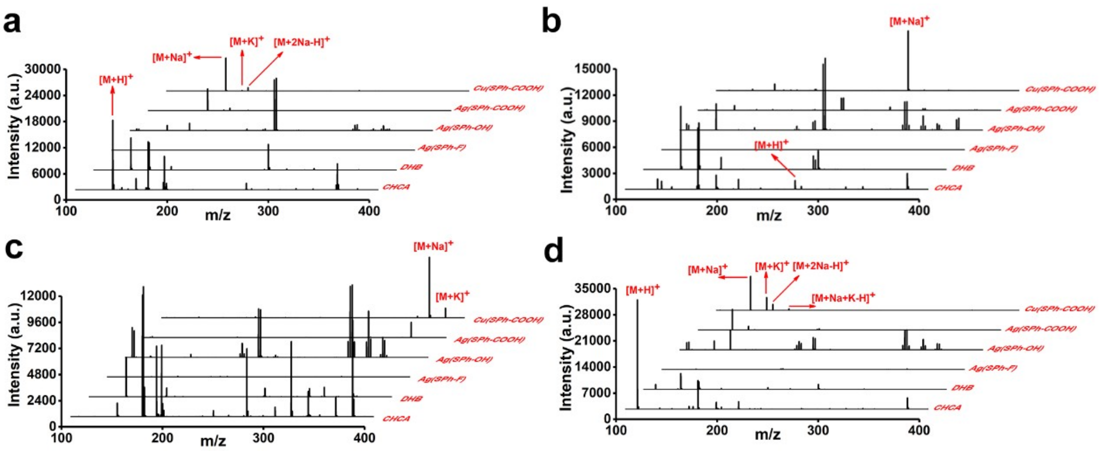

Figure S6. Mass spectrum of (a) HAT, (b) Ado, (c) Malt, (d) C, with different matrixes consisting of four OMCs, CHCA, and DHB.

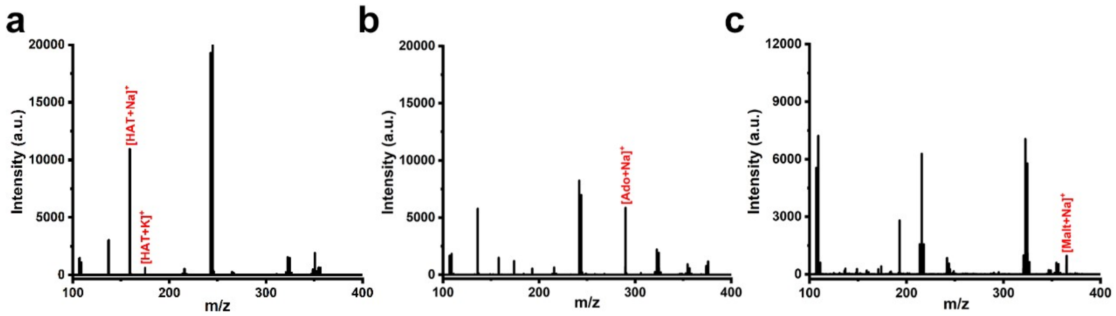

Figure S7. Mass spectrum of (a) HAT, (b) Ado, and (c) Malt with Ag(SPh-COOH) as substrate at a laser intensity of 416 μJ/mm^2.

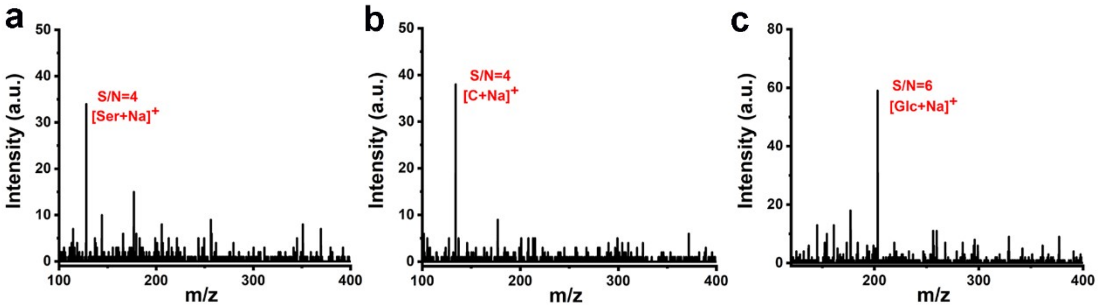

Figure S8. Mass spectra of 200 pg/mL Ser, 200 pg/mL C and 500 pg/mL Glc.

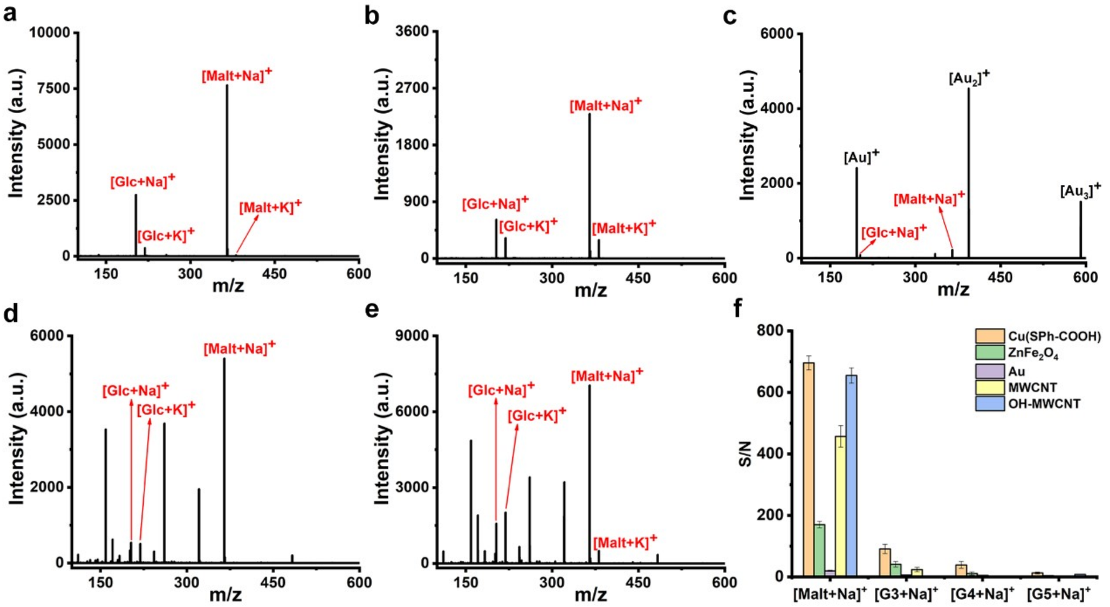

Figure S9. Mass spectrum of Malt with (a) Cu(SPh-COOH), (b) $ZnFe_2O_4$, (c) Au, (d) MWCNT and (e) OH-MWCNT as substrates; (f) the S/N of Malt, G3, G4 and G5 using Cu(SPh-COOH) as substrate with other four nano-substrates as comparison.

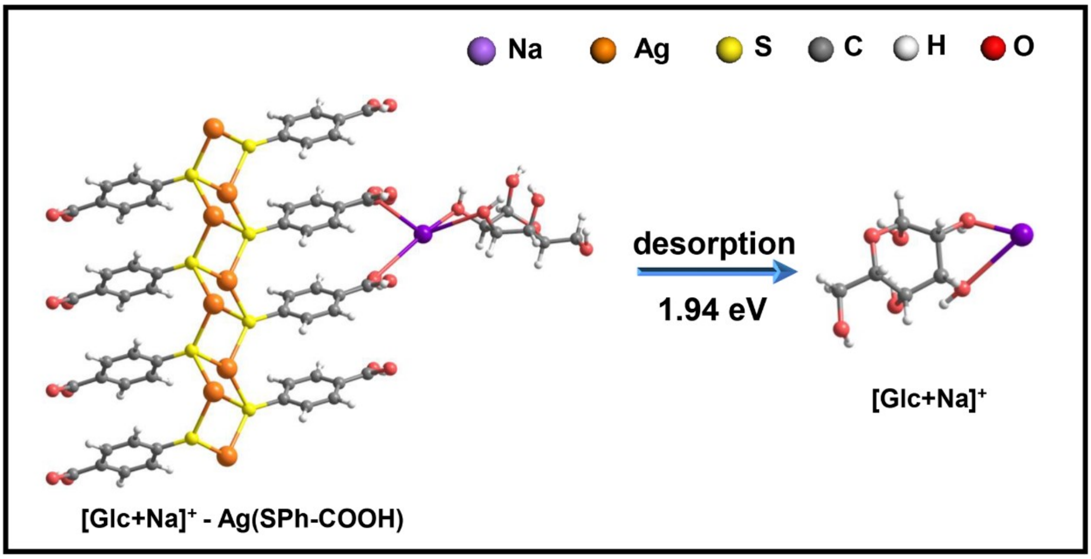

Figure S10. Desorption of $[Glc+Na]^+$ from Ag(SPh-COOH) surface simulated by DFT.

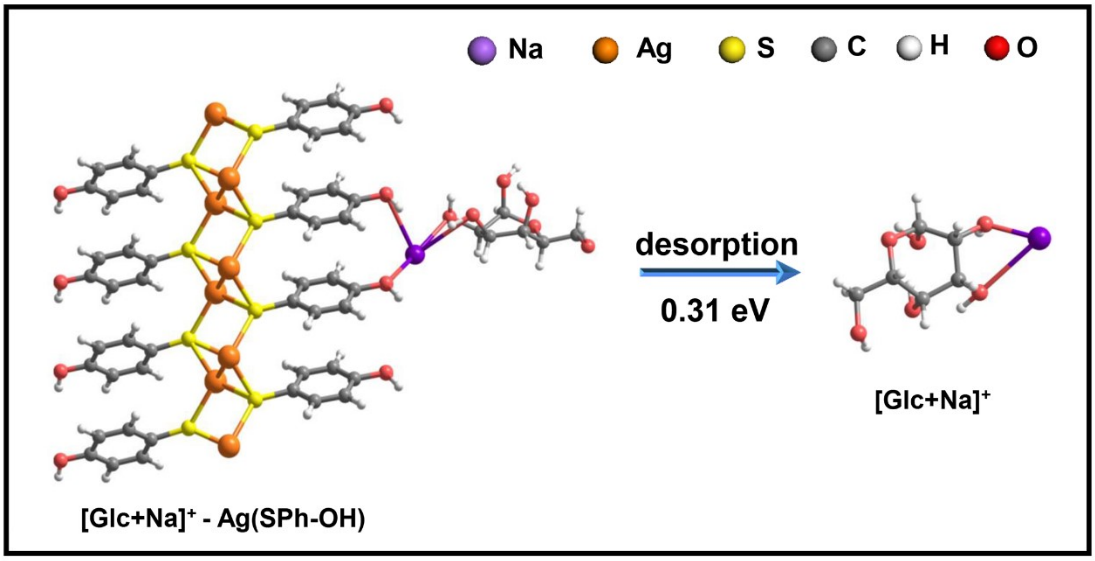

Figure S11. Desorption of $[Glc+Na]^+$ from Ag(SPh-OH) surface simulated by DFT.

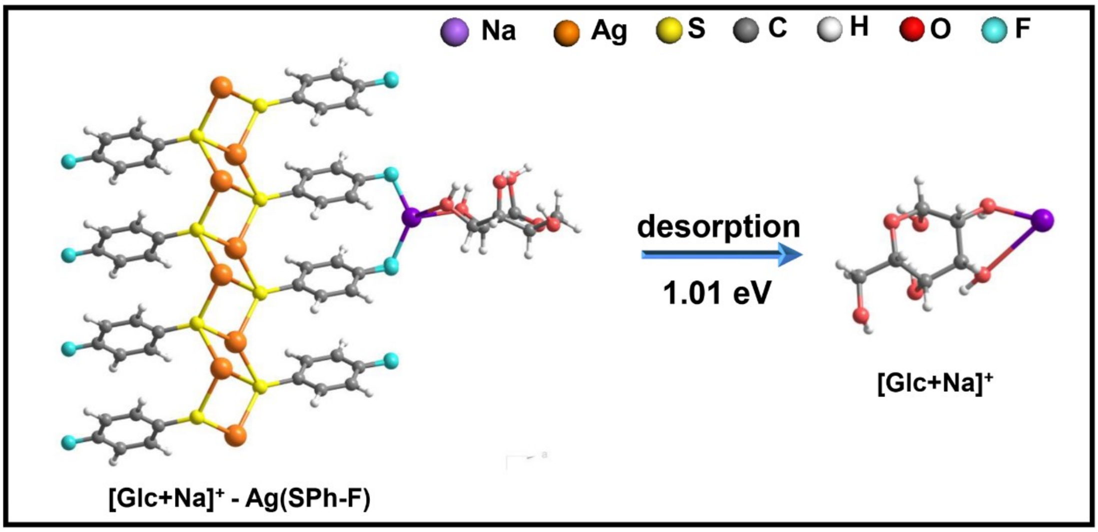

Figure S12. Desorption of $[Glc+Na]^+$ from Ag(SPh-F) surface simulated by DFT.

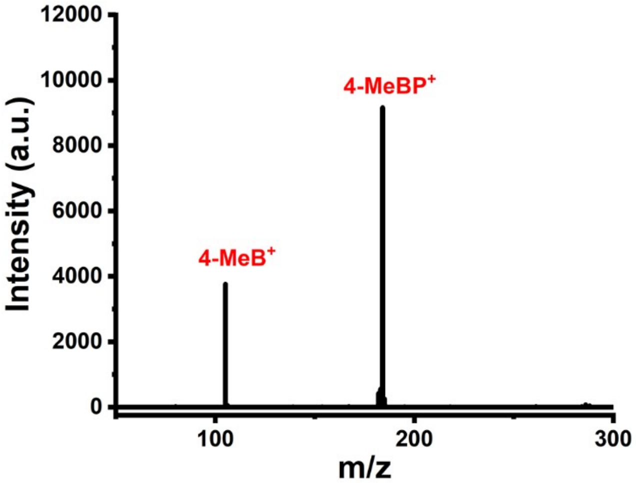

Figure S13. Mass spectrum of 4-MeBP⁺ with Cu(SPh-COOH) as substrate.

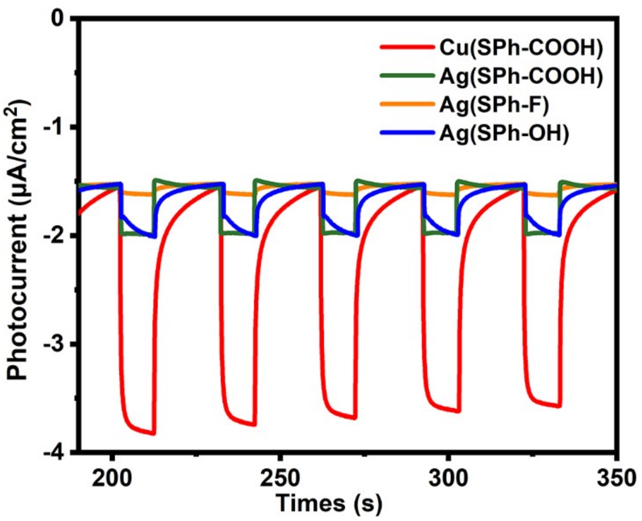

Figure S14. Photocurrent response of the four few-layered OMCs.

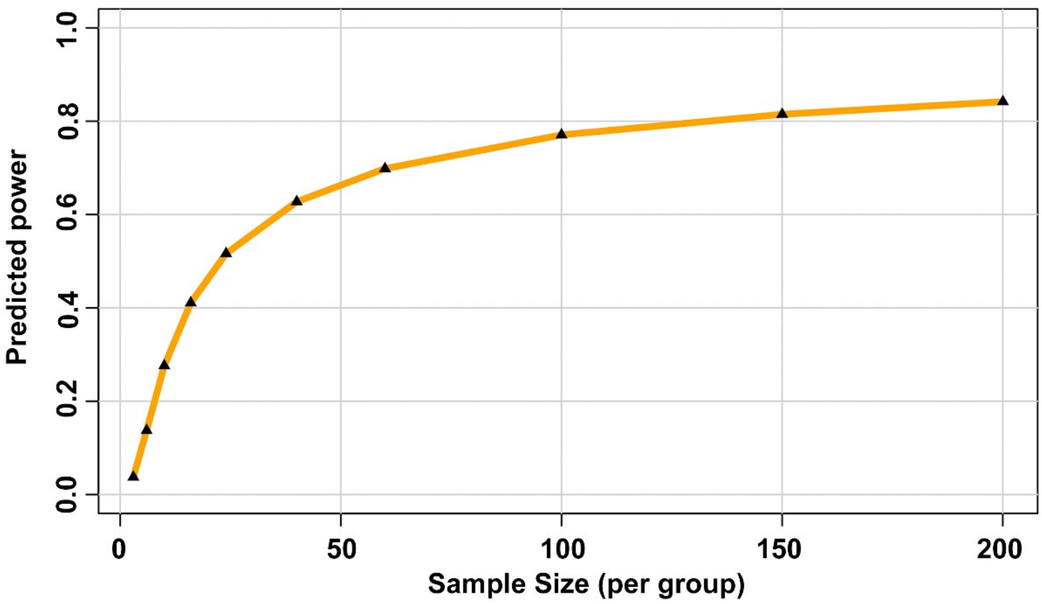

Figure S15. Power analysis of pilot data. The metabolic fingerprinting extracted from 12 samples (6/6 central precocious puberty (CPP)/HC) were analysed to compute the minimum number of samples required with meaningful machine learning.

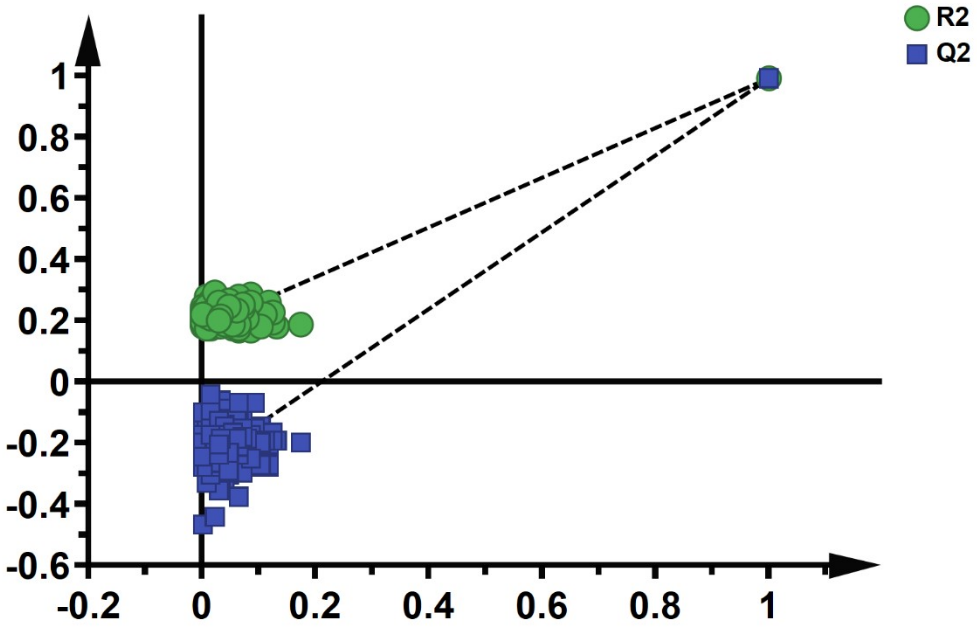

Figure S16. The permutations plot for OPLS-DA of CPP vs HC.

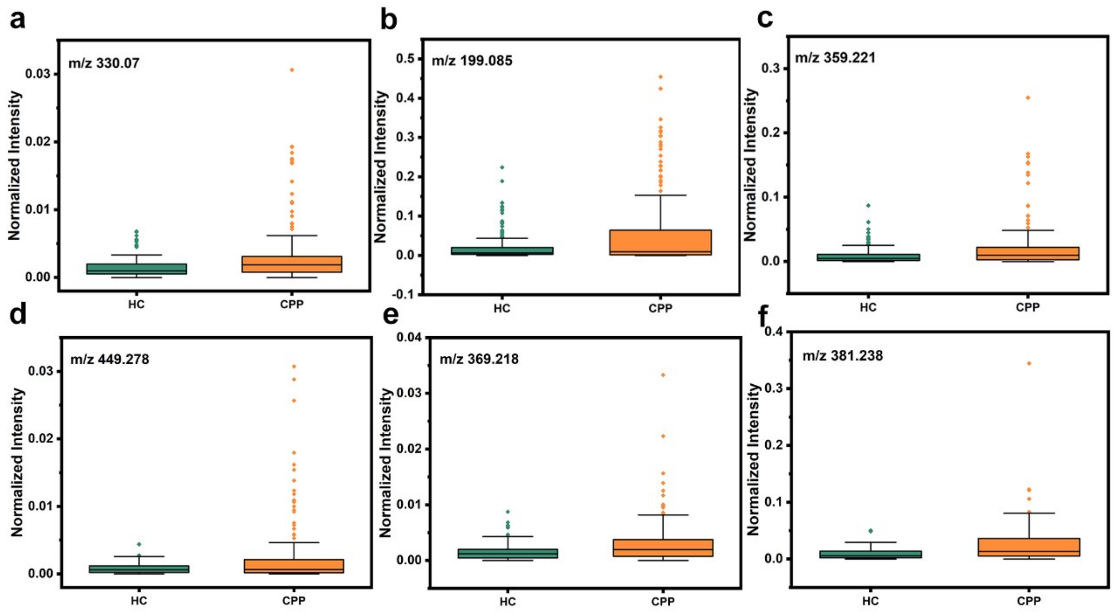

Figure S17. Dysregulation of six potential biomarkers in serum samples between HC and CPP. (a) Glutathione, (b) Serotonin, (c) 15-HPETE, (d) (3beta,22E,24R)-3-Hydroxyergosta-5,8,22-trien-7-one, (e) Docosapentaenoic acid (22n-3), (f) Pregnanetriol.

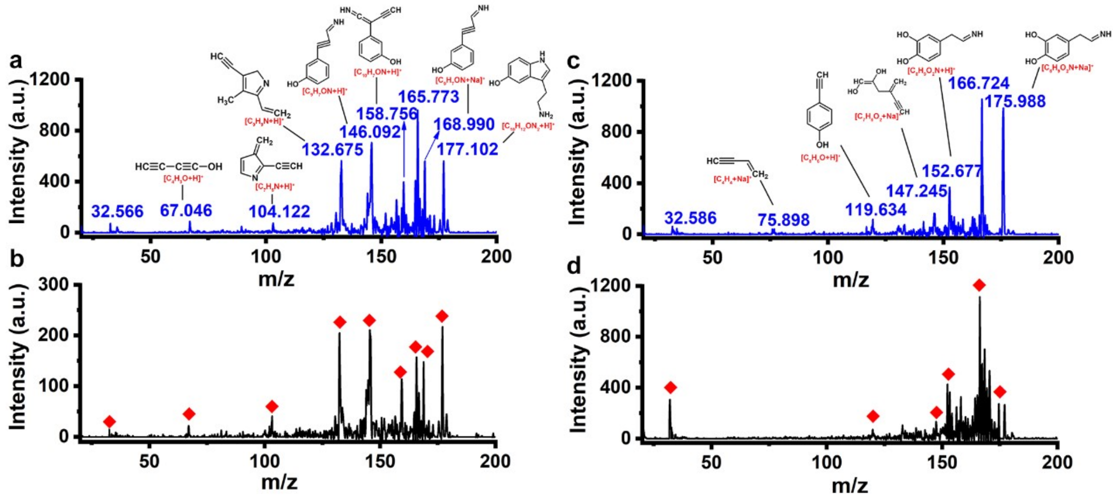

Figure S18. MS/MS results of a) serotonin, b) 199.085 Da in CPP, c) dopamine, and d) 176.069 Da in CPP. Signals of fragment ions as marked were matched with human metabolome database.

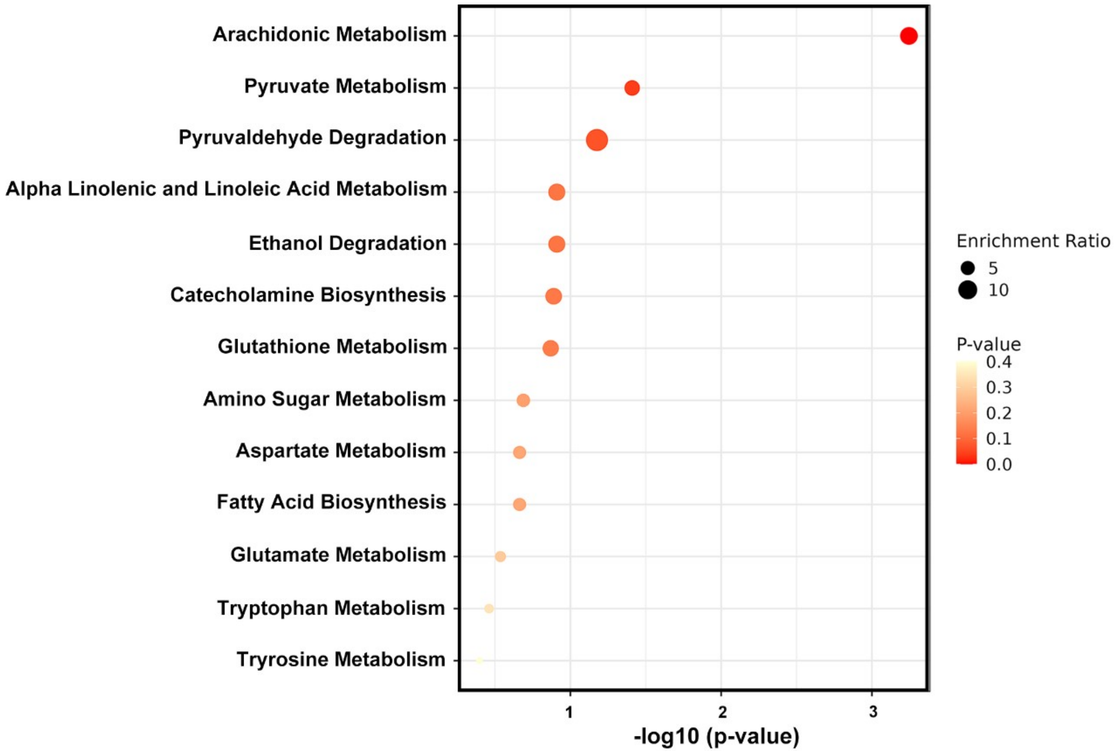

Figure S19. The enrichment of different sets in serum.

Supplementary Tables

Table S1. Demographic information of patients and healthy controls for OPLS-DA.

Subjects	Gender	Age	Treated	Subjects	Gender	Age	Treated
HC	Female	10	NA	CPP	Female	10	NA
HC	Female	9	NA	CPP	Female	10	NA
HC	Female	5	NA	CPP	Female	6	NA
HC	Female	6	NA	CPP	Male	9	NA
HC	Female	12	NA	CPP	Female	11	NA
HC	Female	7	NA	CPP	Female	10	NA
HC	Female	9	NA	CPP	Male	8	NA
HC	Female	6	NA	CPP	Male	12	NA
HC	Female	11	NA	CPP	Female	7	NA
HC	Male	10	NA	CPP	Male	9	NA
HC	Male	6	NA	CPP	Female	14	NA
HC	Female	9	NA	CPP	Female	10	NA
HC	Female	14	NA	CPP	Female	13	NA
HC	Female	6	NA	CPP	Female	7	NA
HC	Female	10	NA	CPP	Female	8	NA
HC	Female	8	NA	CPP	Female	12	NA
HC	Female	13	NA	CPP	Female	10	NA
HC	Female	12	NA	CPP	Female	10	NA
HC	Female	7	NA	CPP	Female	7	NA
HC	Female	9	NA	CPP	Male	10	NA
HC	Female	5	NA	CPP	Female	12	NA
HC	Female	11	NA	CPP	Female	13	NA
HC	Female	10	NA	CPP	Female	10	NA
HC	Male	8	NA	CPP	Female	12	NA
HC	Female	9	NA	CPP	Male	11	NA
HC	Female	12	NA	CPP	Female	15	NA
HC	Female	6	NA	CPP	Female	9	NA
HC	Female	9	NA	CPP	Female	13	NA
HC	Female	13	NA	CPP	Female	6	NA
HC	Female	11	NA	CPP	Female	10	NA
HC	Female	9	NA	CPP	Male	9	NA
HC	Female	10	NA	CPP	Male	6	NA
HC	Male	7	NA	CPP	Female	12	NA
HC	Female	13	NA	CPP	Female	9	NA
HC	Female	13	NA	CPP	Female	13	NA
HC	Female	8	NA	CPP	Female	9	NA
HC	Female	8	NA	CPP	Female	8	NA
HC	Female	6	NA	CPP	Female	10	NA
HC	Male	10	NA	CPP	Female	10	NA

HC	Female	7	NA	CPP	Male	9	NA
HC	Female	15	NA	CPP	Female	9	NA
HC	Female	11	NA	CPP	Female	10	NA
HC	Female	12	NA	CPP	Female	7	NA
HC	Female	9	NA	CPP	Female	10	NA
HC	Female	5	NA	CPP	Male	9	NA
HC	Female	14	NA	CPP	Female	9	NA
HC	Female	7	NA	CPP	Female	7	NA
HC	Female	6	NA	CPP	Male	11	NA
HC	Female	7	NA	CPP	Female	6	NA
HC	Male	6	NA	CPP	Female	9	NA
HC	Female	10	NA	CPP	Male	9	NA
HC	Female	12	NA	CPP	Female	9	NA
HC	Female	8	NA	CPP	Female	9	NA
HC	Male	6	NA	CPP	Female	12	NA
HC	Male	7	NA	CPP	Female	15	NA
HC	Female	6	NA	CPP	Female	10	NA
HC	Female	14	NA	CPP	Female	9	NA
HC	Female	10	NA	CPP	Female	7	NA
HC	Female	9	NA	CPP	Female	7	NA
HC	Female	5	NA	CPP	Female	15	NA
HC	Female	6	NA	CPP	Female	6	NA
HC	Female	12	NA	CPP	Female	10	NA
HC	Female	7	NA	CPP	Female	8	NA
HC	Female	9	NA	CPP	Female	6	NA
HC	Female	4	NA	CPP	Female	12	NA
HC	Female	11	NA	CPP	Female	10	NA
HC	Female	10	NA	CPP	Male	8	NA
HC	Male	6	NA	CPP	Female	8	NA
HC	Female	6	NA	CPP	Female	7	NA
HC	Female	14	NA	CPP	Female	8	NA
HC	Female	6	NA	CPP	Female	7	NA
HC	Male	10	NA	CPP	Female	10	NA
HC	Male	8	NA	CPP	Male	5	NA
HC	Female	13	NA	CPP	Female	10	NA
HC	Female	9	NA	CPP	Female	12	NA
HC	Female	13	NA	CPP	Female	12	NA
HC	Male	11	NA	CPP	Female	8	NA
HC	Female	9	NA	CPP	Male	9	NA
HC	Female	10	NA	CPP	Female	9	NA
HC	Male	7	NA	CPP	Female	10	NA
HC	Female	13	NA	CPP	Male	9	NA
HC	Female	13	NA	CPP	Female	10	NA

HC	Female	8	NA	CPP	Female	10	NA
HC	Male	8	NA	CPP	Female	10	NA
HC	Male	14	NA	CPP	Female	9	NA
HC	Male	10	NA	CPP	Female	9	NA
HC	Female	7	NA	CPP	Female	10	NA
HC	Female	15	NA	CPP	Female	8	NA
HC	Female	11	NA	CPP	Female	11	NA
HC	Male	9	NA	CPP	Female	11	NA
HC	Female	9	NA	CPP	Female	10	NA
HC	Female	5	NA	CPP	Female	9	NA
HC	Female	14	NA	CPP	Female	8	NA
HC	Male	7	NA	CPP	Female	9	NA
HC	Female	4	NA	CPP	Female	9	NA
HC	Female	10	NA	CPP	Female	10	NA
HC	Female	7	NA	CPP	Female	9	NA
HC	Female	15	NA	CPP	Female	10	NA
HC	Female	11	NA	CPP	Female	9	NA
HC	Female	13	NA	CPP	Female	10	NA
HC	Female	9	NA	CPP	Female	12	NA
HC	Female	5	NA	CPP	Female	12	NA
HC	Male	14	NA	CPP	Female	13	NA
HC	Female	7	NA	CPP	Male	11	NA
HC	Female	12	NA	CPP	Female	15	NA
HC	Female	7	NA	CPP	Female	9	NA
HC	Female	6	NA	CPP	Female	9	NA
HC	Male	10	NA	CPP	Female	10	NA
HC	Female	7	NA	CPP	Male	12	NA
HC	Female	13	NA	CPP	Female	9	NA
HC	Female	13	NA	CPP	Female	11	NA
HC	Female	8	NA	CPP	Male	10	NA
HC	Male	8	NA	CPP	Female	9	NA
HC	Female	4	NA	CPP	Female	8	NA
HC	Male	10	NA	CPP	Female	9	NA
HC	Female	10	NA	CPP	Female	9	NA
HC	Female	7	NA	CPP	Female	10	NA
HC	Female	15	NA	CPP	Female	9	NA
HC	Female	11	NA	CPP	Female	10	NA
HC	Female	10	NA	CPP	Female	9	NA
HC	Male	9	NA	CPP	Female	10	NA
HC	Female	5	NA	CPP	Female	9	NA
HC	Female	14	NA	CPP	Female	10	NA
HC	Female	6	NA	CPP	Female	10	NA
HC	Female	10	NA	CPP	Female	10	NA

HC	Male	8	NA	CPP	Female	9	NA
HC	Female	8	NA	CPP	Male	9	NA
HC	Female	6	NA	CPP	Male	10	NA
HC	Female	7	NA	CPP	Female	6	NA
HC	Female	6	NA	CPP	Female	10	NA
HC	Female	14	NA	CPP	Female	8	NA
HC	Male	10	NA	CPP	Female	6	NA
HC	Female	9	NA	CPP	Male	12	NA
HC	Female	5	NA	CPP	Female	10	NA
HC	Female	6	NA	CPP	Female	8	NA
HC	Male	12	NA	CPP	Female	8	NA
HC	Female	7	NA	CPP	Female	7	NA
HC	Male	11	NA	CPP	Female	8	NA
HC	Female	8	NA	CPP	Male	15	NA
HC	Female	13	NA	CPP	Female	10	NA
HC	Female	14	NA	CPP	Female	9	NA
HC	Female	14	NA	CPP	Female	7	NA
HC	Female	15	NA	CPP	Female	11	NA
HC	Female	9	NA	CPP	Female	6	NA
HC	Male	13	NA	CPP	Male	9	NA
HC	Female	13	NA	CPP	Female	9	NA
HC	Female	8	NA	CPP	Male	10	NA
HC	Female	11	NA	CPP	Female	7	NA
HC	Male	7	NA	CPP	Female	10	NA
HC	Female	10	NA	CPP	Female	9	NA
HC	Female	11	NA	CPP	Female	9	NA
HC	Female	11	NA	CPP	Female	8	NA
HC	Male	9	NA	CPP	Male	10	NA
HC	Female	10	NA	CPP	Female	6	NA
HC	Female	5	NA	CPP	Female	7	NA
HC	Female	8	NA	CPP	Female	13	NA
				CPP	Female	10	NA

Table S2. Identification of the metabolites.

m/z	Addition signal	VIP	FDR	P value	Metabolite	HMDB	Δppm
104.993	$[M+2Na-H]^+$	2.124	0.00145	3.6535E-5	Acetic acid	HMDB0000042	7
176.069	$[M+Na]^+$	2.181	6.4534E-4	8.9948E-6	Dopamine	HMDB0000073	5
193.007	$[M+2Na-H]^+$	2.285	5.0007E-4	5.6217E-6	Citramalic acid	HMDB0000426	7
199.085	$[M+Na]^+$	2.128	8.3735E-4	1.4548E-5	Serotonin	HMDB0000259	4
330.07	$[M+Na]^+$	2.093	0.00118	2.5047E-5	Glutathione	HMDB0000125	9
349.213	$[M+2Na-H]^+$	2.475	1.1063E-4	3.9572E-7	Arachidonic acid	HMDB0001043	5
353.232	$[M+Na]^+$	2.908	3.921E-6	2.0036E-9	9,10,13-TriHOME	HMDB0004710	6
359.221	$[M+Na]^+$	1.852	0.00391	1.8537E-4	15-HPETE	HMDB0000191	6
369.218	$[M+K]^+$	2.263	6.9595E-4	1.0778E-5	Docosapentaenoic acid (22n-3)	HMDB0001976	3
381.238	$[M+2Na-H]^+$	2.749	1.9587E-5	2.0017E-8	Pregnanetriol	HMDB0006070	1
397.198	$[M+2Na-H]^+$	2.346	5.0007E-4	4.5282E-6	Prostaglandin E2	HMDB0001220	5
449.278	$[M+K]^+$	2.082	0.00183	4.7711E-5	(3beta,22E,24R)-3-Hydroxyergosta-5,8,22-trien-7-one	HMDB0032106	8

The p value was obtained from the t-test of single m/z signal between diseases and controls.

References

1 Y. Li, X. Jiang, Z. Fu, Q. Huang, G. E. Wang, W. H. Deng, C. Wang, Z. Li, W. Yin, B. Chen, G. Xu, *Nat. Commun.* 2020, **11**, 261.
2 Y.-H. Cheng, K.-M. Ng, *Anal. Chem.* 2020, **92**, 5645-5649.
3 J. Hutter, M. Iannuzzi, F. Schiffmann, J. VandeVondele, *Wiley Interdiscip. Rev.: Comput. Mol. Sci.* 2014, **4**, 15–25.
4 B.G. LIPPERT, J. HUTTER, M. PARRINELLO, *Mol. Phys.* 1997, **92**, 477–488.
5 J. VandeVondele, J. Hutter, *J. Chem. Phys.* 2007, **127**, 114105.
6 S. Goedecker, M. Teter, J. Hutter, *Phys. Rev. B.* 1996, **54**, 1703–1710.
7 S. Grimme, J. Antony, S. Ehrlich, H. Krieg, *J. Chem. Phys.* 2010, **132**, 154104.
8 J. Yang, X. Yin, L. Zhang, X. Zhang, Y. Lin, L. Zhuang, W. Liu, R. Zhang, X. Yan, L. Shi, W. Di, L. Feng, Y. Jia, J. Wang, K. Qian, X. Yao, Adv. Mater. 2022, **34**, 2201422.
